# Device associated –health care associated infections monitoring, prevention and cost assessment at intensive care unit of University Hospital in Poland (2015–2017)

**DOI:** 10.1186/s12879-020-05482-w

**Published:** 2020-10-16

**Authors:** Wieslawa Duszynska, Victor Daniel Rosenthal, Aleksander Szczesny, Katarzyna Zajaczkowska, Michal Fulek, Jacek Tomaszewski

**Affiliations:** 1 Department and Clinic of Anaesthesiology and Intensive Therapy, Wroclaw Medical University, L.Pasteura Street 1, 50-367 Wroclaw, Poland; 2 International Nosocomial Infection Control Consortium, Buenos Aires, Argentina; 3 The Students Scientific Association by Department and Clinic of Anaesthesiology and Intensive Therapy, Wroclaw, Poland

**Keywords:** Health care associated infections, DA-HAIs, ISOS, Length of stay, Bundle, ICU

## Abstract

**Background:**

Device-associated health care-associated infections (DA-HAIs) in intensive care unit (ICU) patients constitute a major therapeutic issue complicating the regular hospitalisation process and having influence on patients’ condition, length of hospitalisation, mortality and therapy cost.

**Methods:**

The study involved all patients treated > 48 h at ICU of the Medical University Teaching Hospital (Poland) from 1.01.2015 to 31.12.2017. The study showed the surveillance and prevention of DA-HAIs on International Nosocomial Infection Control Consortium (INICC) Surveillance Online System (ISOS) 3 online platform according to methodology of the INICC multidimensional approach (IMA).

**Results:**

During study period 252 HAIs were found in 1353 (549F/804M) patients and 14,700 patient-days of hospitalisation. The crude infections rate and incidence density of DA-HAIs was 18.69% and 17.49 ± 2.56 /1000 patient-days. Incidence density of ventilator-associated pneumonia (VAP), central line-associated bloodstream infection (CLA-BSI) and catheter-associated urinary tract infection (CA-UTI) per 1000 device-days were 12.63 ± 1.49, 1.83 ± 0.65 and 6.5 ± 1.2, respectively. VAP(137) constituted 54.4% of HAIs, whereas CA-UTI(91) 36%, CLA-BSI(24) 9.6%.The most common pathogens in VAP and CA-UTI was multidrug-resistant (MDR) *Acinetobacter baumannii* (57 and 31%), and methicillin-resistant *Staphylococcus epidermidis* (MRSE*)* in CLA-BSI (45%). MDR Gram negative bacteria (GNB) 159 were responsible for 63.09% of HAIs. The length of hospitalisation of patients with a single DA-HAI at ICU was 21(14–33) days, while without infections it was 6.0 (3–11) days; *p* = 0.0001. The mortality rates in the hospital-acquired infection group and no infection group were 26.1% vs 26.9%; *p* = 0.838; OR 0.9633;95% CI (0.6733–1.3782). Extra cost of therapy caused by one ICU acquired HAI was US$ 11,475/Euro 10,035. Hand hygiene standards compliance rate was 64.7%, while VAP, CLA-BSI bundles compliance ranges were 96.2–76.8 and 29–100, respectively.

**Conclusions:**

DA-HAIs was diagnosed at nearly 1/5 of patients. They were more frequent than in European Centre Disease Control report (except for CLA-BSI), more frequent than the USA CDC report, yet less frequent than in limited-resource countries (except for CA-UTI). They prolonged the hospitalisation period at ICU and generated substantial additional costs of treatment with no influence on mortality. The *Acinetobacter baumannii MDR* infections were the most problematic therapeutic issue. DA-HAIs preventive methods compliance rate needs improvement.

## Background

Monitoring hospital infections is one of the most important elements in the prevention and control of device-associated health care-associated infections (DA-HAIs). Ss shown in scientific literature, monitoring can lead to DA-HAIs reduction if implemented with a multidimensional approach [1, 2]. Hospital infections, which complicate the regular hospitalisation process, are a major therapeutic issue leading to compromising patients’ condition (sometimes increased mortality), prolonged treatment periods and increased hospitalisation costs [3, 4].^,^Intensive care unit (ICU) nosocomial infections are more commonly associated with invasive treatment and diagnostic techniques as well as using life-supporting or monitoring devices directly or indirectly. Nevertheless, infection risk factors were also found to be present on admission [5, 6]. According to published data, over 50% of ICU patients are infected [7, 8]. It was also found that DA-HAIs concern about 24.3–27.6% of ICU patients [6, 9]. Additionally, geographic region, country income and hospital type influence the frequency of DA-HAIs worldwide [10–14]. Monitoring of hospital infections in Poland was initiated by the Polish Society of Hospital Infections in 1999 and remains effective due to their cooperation with the European Centre for Disease Prevention and Control (ECDC) [15–17]. Even though Poland has been developing its infection control system quickly and effectively, few data has been published in English on hospital-acquired infections in patients treated at Polish ICUs [8, 9, 15–17].

The International Nosocomial Infection Control Consortium (INICC) network operates by means of an online surveillance system - the INICC Surveillance Online System (ISOS) 3- and a systematic multidimensional approach - the INICC Multidimensional Approach (IMA) - whose effectiveness for reduction of DA-HAI rates has been shown in the scientific literature [18–23]. The IMA is a system aimed to measure and reduce DA-HAI rates, mortality, LOS, costs, bacterial resistance and antibiotic consumption that comprises the simultaneous implementation of 6 components: (1) bundles, (2) education and training, (3) online outcome surveillance of DA-HAI rates and their adverse consequences; (4) online process surveillance to evaluate compliance with bundles; (5) online feedback of DA-HAI rates and their adverse consequences; and (6) online performance feedback. The ISOS applies the definitions of DA-HAIs developed by the Centre for Disease Prevention and Control /National Healthcare Safety Network (CDC/NHSN) and standardized methodologies, thereby promoting applied research and evidence-based infection prevention practices [1]. ISOS, implemented in more than 700 ICUs in 66 countries (predominantly developing and underfunded), has proven to be a very useful tool in DA-HAIs surveillance [1, 11].

The aim of this study is to show the results of active prospective surveillance and monitoring of infections, as well as to evaluate the compliance with preventive guidelines using INICC ISOS3 platform modules: Surveillance of HAIs-Full data-Adults and Paediatric ICU and Monitoring Infection Control Practices including Monitoring Compliance with Hand Hygiene (HH), Monitoring Compliance of ventilator-associated pneumonia (VAP), central line-associated bloodstream infection (CLA-BSI), and catheter-urinary tract infection (CA-UTI) Prevention Bundle.

## Methods

### Data collection

The observational prospective study involved 1353 patients treated at Wroclaw Medical University Teaching Hospital in their ICU from 1/01/2015 to 31/12/2017. Preventive guidelines were evaluated in the last year of the observational period. The study protocol was approved by the Bioethics Committee of Wroclaw Medical University (No: KB-605/2016) in accordance with the Declaration of Helsinki. All analysed data (including laboratory results obtained from the patients during routine patients care and infections monitoring) entered into ISOS was previously anonymised and a statement covering patients data confidentiality was fully respected during manuscript preparation. In consequence, no consent or statement was needed. Consent from the local Institutional Bioethics Committee also included approval for publication of the data. Data concerning DA-HAIs was compared with the results of international reports of NHNS [10], INICC [24], ECDC [25] and our own earlier study.

The following data was entered and collected on the ISOS3 platform: age, sex, reason of hospitalisation, use of respirator support in patients with a tracheal tube or a tracheotomy tube, use of catheter in central line as well as urinary catheters, time of admission and discharge from the ICU, result of treatment, date of diagnosis and clinical presentation of a hospital infection and following particular guidelines in the prevention of infections called VAP, CA-UTI, CLA-BSI “bundles” as well as following hand hygiene standards.

### Clinical and microbiological diagnosis of DA-HAIs

DA-HAIs were diagnosed in patients hospitalised > 48 h, based on clinical symptoms, infection markers [C-reactive protein (CRP), procalcitonin (PCT), leukocytosis], diagnostic imaging (chest X-ray) and microbiological test results (blood, tracheal aspirate, central venous catheter tip, urine) according to CDC/INICC guidelines and their yearly updates [1, 26]. All materials submitted for microbiological analysis were sampled and assessed qualitatively and quantitatively according to accepted European Union standards. Microbiological diagnoses were performed at University Hospital Microbiology Laboratory which implemented and applied the recommendation of the European Committee on Antimicrobial Susceptibility Testing (EUCAST) [27]. Additionally, susceptibility of microorganisms with Minimal Inhibitory Concentration (MIC) and resistance mechanisms assessment were determined and interpreted in accordance with the applicable recommendations of EUCAST [27]. A highly multiplexed, rapid point-of-care PCR test for respiratory pathogens and blood pathogens (FILMARRAY respiratory Panel and Blood Culture Identification Panel, BioFire Diagnostics USA) were used in early microbiological diagnosis. Two doctors working at the ICU and a microbiologist were involved in diagnosing the infection.

### Epidemiological indicators

Epidemiological indicators were assessed as follow: DA-HAIs incidence density = number of DA-HAIs/ 1000 patient-days; VAP incidence density = number of VAP/ 1000 mechanical ventilator (MV)-days; CLA-BSI incidence density = number of CLA-BSIs/ 1000 central line (CL)-days; UTI incidence density = number of UTIs/ 1000 urinary catheter (UC)-days; Infections rate (%) = number of infections/ number of patients hospitalised in a given time frame; Device utilisation ratio (DU-R) = number of days of MV, CL or UC/ number of patient-days in a given time frame × 100 [1].

Additionally, a percentage of specific clinical presentations of infections among the general number of HAIs was assessed, and an analysis of pathogens causing particular clinical presentations of infections and multidrug resistance assessment was conducted.

### Compliance with HAIs preventive bundles and hand hygiene standard

The study analyses data concerned with compliance with VAP, CA-UTI, CLA-BSI preventive “bundles”, which were entered into the system at least once per week, showing a percentage of compliance with particular components of the preventive guidelines. Components of INICC Infection Control Bundle for VAP prevention included the following elements: 30–50 degree elevation of patients head, performed assessment of readiness to wean, subglottic suctioning, endotracheal cuff pressure of at least 20 cm, comprehensive oral care with an antiseptic solution, condensate in ventilatory circuits, gastric over- distention, stress ulcer prophylaxis, deep vein thrombosis prophylaxis. Components of INICC bundle for UTI prevention are as follows: maximal barriers precautions when catheter was inserted, single-use lubricant used, sterile closed drainage system, urinary catheter never disconnected, urinary catheter above the leg, urinary collecting bag below the level of bladder, urinary collecting bag with less than 75% of capacity full. Components of INICC bundle for CLA-BSI prevention are as follows: hand hygiene compliance before catheter insertion or manipulation, maximum sterile precaution barrier during insertion, chlorhexidine skin antisepsis, daily assessment of the need of catheter, presence of sterile dressing, type of dressing, good condition of dressing, single use flushing, type of set connector, type of bag container for intravenous infusion, administration equipment date, daily bath with a 2% chlorhexidine-impregnated washcloth.

Moreover, the anonymous study assessed the baseline HH compliance rate by healthcare workers at ICU at a different work times every 3 months. Five moments for hand hygiene were checked: before patient contact, before aseptic procedures, after patient contact, after body fluid exposition risk, after contact with patient surroundings. Staff was aware of the checks, yet not specifically of when the checks were carried out. The study showed the percentage of staff following hand hygiene standards.

### Economic cost analysis based on a prolonged LOS

As an economic cost analysis, the study compared the length of stay (LOS) in ICU patients with a single infection, as well as with multiple infections, with the LOS of patients without hospital infections. Based on a prolonged LOS (extra LOS) and the patient-day cost an additional cost resulting from hospital infections was calculated [1]. Total daily cost per one ICU patient was retrieved from the Financial Management of the hospital. Components of this costs are included as follows: human resources, medications, consumables, laboratory tests, investigation tests, administrative expenses assigned to ICU. Total daily cost per one ICU patient was assessed once a year. The hospital did not perform a detailed and statistical cost analysis per one individual patient as well as for infected and not infected patient. Our study used mean values of these costs between the years 2015 and 2017, which was €669 = US$ 765. Total daily cost was entered into ISOS calculator in order to obtain cost analysis.

### Statistical analyses

Statistical analyses were performed using the STATISTICA program version 13.1(StatSoft Inc., Tulsa, USA) Data are presented as mean values ± standard deviation (SD) or median and interquartile range (IQR). Descriptive statistics were computed for all study variables. Discrete variables are expressed as counts and percentage or median and IQR. Distribution of qualitative variables were analysed using Chi-square test, Person’s chi-squared test, the Mann-Whitney U test which were used adequately to the strength of the group. For multiple comparisons was also used the Kruskal- Wallis ANOVA test with post hoc analysis and Fieller test. *P* < 0.05 was considered as statistically significant.

## Results

**Fig. 1:**
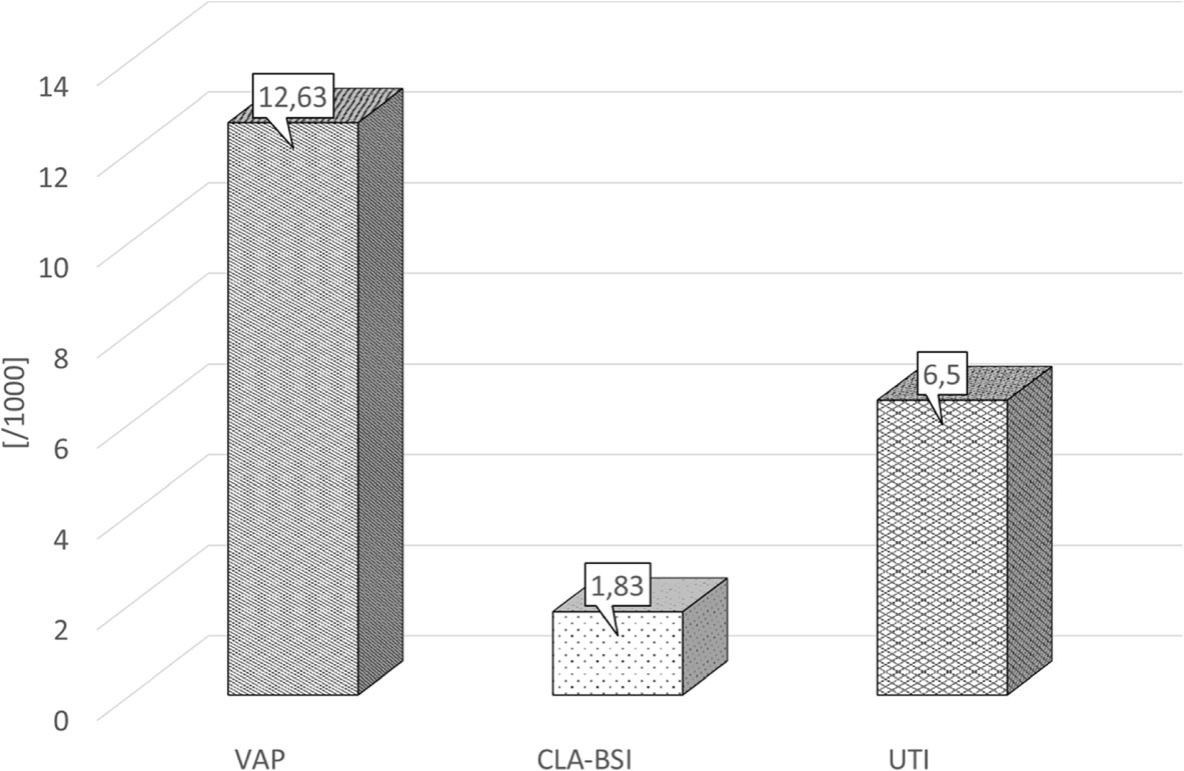
VAP, CLA-BSI and UTI mean incidence density/1000 DU-R. Abbreviations: UTI, catheter-associated urinary tract infection; CLA-BSI, central line-associated bloodstream infection; DU-R, device utilization ratio; VAP, ventilator-associated pneumonia

Among the 1353 patients over the 14,700 patient-day hospitalisation period, 252 hospital infections were diagnosed. See Table 1 for patients’ characteristics. DA-HAIs incidence density [mean ± SD/median (IQR)] was 17.49 ± 2.56 /17.13(16.13–18.68)/1000 patient-days. The crude infections rate of DA-HAIs was 18.62%. VAP was the most commonly diagnosed DA-HAI found in 137 patients (54.4%), followed by CA-UTI in 91 patients (36%) and CLA-BSI at 24 patients (9.6%).VAP incidence density [(median (IQR)]/1000 MV-days was 13.66 (12.01–13.77),whereas UTI 5.85(5.8–6.8)/1000 UC-days and CLA-BSI 1.63(1.47–2.09)/1000 CL-days. See Fig. 1 for VAP, UTI, CLA-BSI mean incidence density/ 1000 DU-R.

**Table 1:** Patient characteristics. Data is showed as numerical values, percentage values, median values (IQR), 95% CI

Total number of patients, n	1353
Female, n(%)	549(40.56)
Male, n(%)	804(59.44)
Age years, (%)	
79–91	17.48
65–78	32.03
52–64	23.9
39–51	10.63
Medical patients, n(%)	566(41.82)
Surgical patients, n(%)	787(57.9)
Total patient-days, n	14,700
Total mechanical ventilation –days, n	11,310
DU-R mechanical ventilation, (%)	76.41
Total urinary catheter days, n	13,039
DU-R urinary catheter, (%)	88.75
Total central vascular catheter days, n	14,198
DU-R central vascular catheter, (%)	96.74
Total number died, n(%)	453 (33.5)
LOS in ICU-days, n (IQR)	7(3–15),95% CI(11.13–12.65)

Abbreviations: *CI* confidence interval range; *DU-R* device utilization ratio; *ICU* intensive care unit; *IQR* interquartile range; *LOS* length of stay

**Fig. 2:**
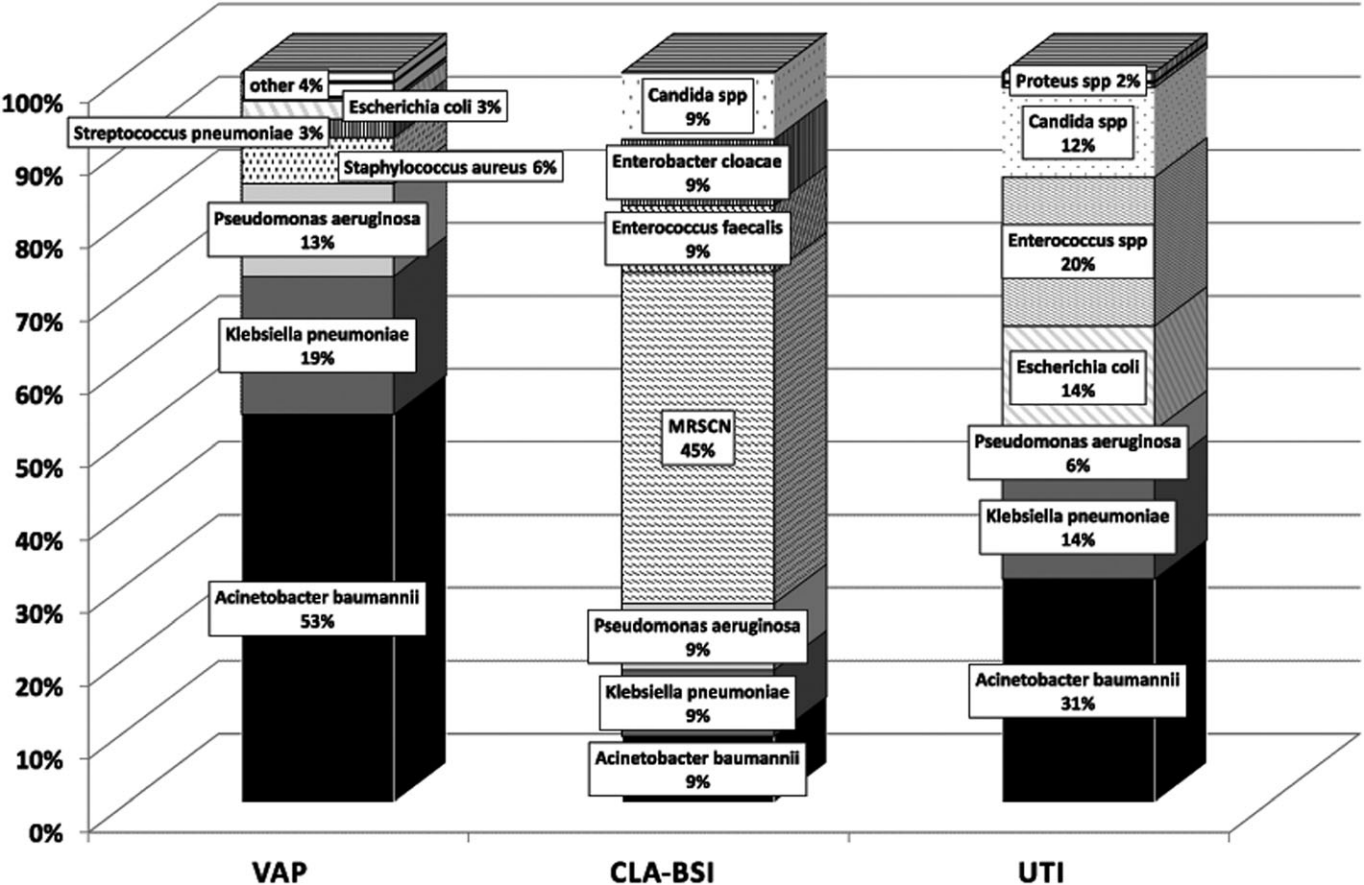
Pathogens responsible for different clinical presentations of DA-HAIs. Abbreviations: UTI, catheter-associated urinary tract infection; CLA-BSI, central line-associated bloodstream infection; DA-HAI, device-associated health care-associated infections; VAP, ventilator-associated pneumonia

The most common pathogen in VAP and UTI was *Acinetobacter baumannii* MDR, comprising 53 and 31% of the general number of pathogens, respectively. The most common etiologic agents of CLA-BSI were methicillin-resistant coagulase-negative staphylococci (MRCNS) strains comprising 45%. See Fig. 2 for a detailed compilation of pathogens of different clinical presentations of DA-HAIs. During the study period Gram Negative Bacteria (GNB) were predominant 229(74. 6%), whereas Gram Positive Bacteria (GPB) 62(20.2%) and fungi 16(5.2%) were found less frequently. MDR GNB were responsible for 159 (63.09%) of HAIs.

Mortality rate in patients with and without hospital infections was similar 26.1% vs 26.9% (*p* = 0.838). HAIs in ICU patients have no significant influence on mortality risk at ICU (p = 0.838/OR 0.9633;CI 0.6733–1.3782). Yet, one hospital infection acquired at ICU tripled the median LOS in comparison to the median LOS of patients with no infections (*p* < 0.0001/95%CI 4.011–5.646). In patients with multiple infections, the median LOS at ICU increased sixfold (p < 0.0001/95%CI 2.532–3.304). See Table 2 for the analysis of how DA-HAIs affected the length of ICU hospital stay and generated additional costs of treatment. See Table 3 for a comparative analysis of the incidence density of particular clinical presentations of hospital infections and data collected in international registers and own study [10, 24, 25, 28]. During the study period 960 opportunities for HH were checked and 154 visits were made at ICU for HAIs preventive bundle assessment. Baseline HH compliance of healthcare workers at ICU was 64.66%, whereas compliance with components of HAIs bundles were assessed only separately. See Table 4 for juxtaposition profile compliance with VAP, CLA-BSI, CA-UTI and hand hygiene preventive bundles.

**Table 2:** Influence of device-associated health care-associated infections on length of stay and additional cost of therapy. Data is showed as numerical values, median values (IQR), 95% CI

	Average LOS of patient with HAIs, n (IQR),95%CI	Average LOS of patient with no HAIs n (IQR),95%CI	Extra LOS of patient with HAIs, n	Extra cost of therapy caused by HAIs
One infection	21(14–33),95%CI(27.72–28.7)	6.0 (3–11),95%CI(8.27–9.42)	15	US$ 11,475 € 10,035
Multiple infections	39.5(31–51),95%CI(35.68–49.41)	6.0 (3–11),95%CI(8.27–9.42)	33.5	US$ 25,627 € 22,411.5
VAP	27(15–41),95%CI(26.77–34.39)	6.0 (3–11),95%CI(8.27–9.42)	21	US$ 16,065 € 14,049
CLA-BSI	30(20–43),95%CI(23.13–38.59)	6.0 (3–11),95%CI(8.27–9.42)	24	US$ 18,360 € 16,056
CA-UTI	29.00(14.5–43.5),95%CI(26.69–35.76)	6.0 (3–11),95%CI(8.27–9.42)	23	US$ 17,595 € 15,387
Additional cost of therapy in observed period				US$ 1413,480 /1 year € 1236,378 /1 year

Legend: real mean value cost of one patient-day was € 669 = US$ 765

Abbreviations: *CA-UTI* catheter-associated urinary tract infection; *CI* confidence interval; *CLA-BSI* central line-associated bloodstream infection; *DA-HAI* device-associated health care-associated infections; *IQR* interquartile range; *LOS* length of stay; *n* number of patients with infections or number of days; *VAP* ventilator-associated pneumonia

**Table 3:** Comparison of incidence density rate of DA- HAIs with data from international registers INICC (2010–15), NHSN (2013), ECDC (2017) and own surveys

	VAP	CAUTI	CLA-BSI
ICU Wroclaw UH HELICS [28]	18.2(15.5–21.6)	4.8(3.5–6.5)	4.01(2.8–5.6)
NHSN [10]	0.9(0.8–1.0)*	1.7(1.6–1.8)*	0.8(0.8–0.9)*
ECDC [25]	9,5(2.5–20.4)**	3.6(0.0–5.0)	3.7(0.7–4.7)
INICC [24]	14.1(13.8–14.4)*	5.1(5.0–5.2)*	5,05(4.9–5.2)*
ICU Wroclaw UH (2015–2017)	12.63(12.01–13.77)	6.5(5.8–6.8)	1.83(1.47–2.08)

Legend:*Data concern to medical –surgical ICU; Data is showed as mean values, percentile values/95%CI confidence interval or minimal and maximal values **

Abbreviations: *CA-UTI* catheter-associated urinary tract infection; *CLA-BSI* central line-associated bloodstream infection; *ECDC* European Centre for Disease Prevention and Control; *ICU* intensive care unit; *INICC* International Nosocomial Infection Control Consortium; *HELICS* The Hospitals in Europe Link for Infection Control through Surveillance; *NHSN* US National Healthcare Safety Network; *UH* University Hospital; *VAP* ventilator-associated pneumonia;

**Table 4:** Compliance with ventilator-associated pneumonia, central line-associated bloodstream infection, catheter-associated urinary tract infection and hand hygiene preventive bundles

VAP		CA-UTI		CLA-BSI		Hand Hygiene	
Elements of bundle	Compliance with bundle	Elements of bundle	Complaince with bundle	Elements of bundle	Compliance with bundle	Elements of bundle	Compliance with bundle
1. 30–50 elevation of head	96,2%	1. Catheter needed	96%	1. Maximal precaution barriers	82%	1. General compliance	64,66%
2. 1. Mechanical ventilator use	96,2%	2. Maximal barriers precautions when inserted	83%	2. Chlorhexidine skin antisepsis	82%	2.Stratified by health workers	Medical doctors -consultants: 100%
							Medical doctors-residents: 83%
3. Type of invasive ventilation1.	Orotracheal: 49,5% Tracheostomy: 50,5%	3. Single-use lubricant used	83%	3. Insertion place and type of catheter	Yugular: 71% Subclavian: 29%		Nurse 67%
							Housekeeper:50%
4. Performed assessments of readiness to wean	77,2%	4. Presence of securement of the catheter	100%	4. Catheter is necessary	97%	3.Stratified by genders	Male: 81%
5. Subglottic suctioning	70,2%	5. Sterile closed drainage system	100%	5. Presence of sterile dressing	100%		
6. Endotracheal cuff pressure of at least 20 cm	65,6%	6. Urinary catheter never disconnected	91,25%	6. Type of dressing:	Sterile transparent dressing: 93% Sterile gauze: 7%		Female: 64%
7. Comprehensive oral care, with an antiseptic solution	91%	7. Urinary catheter above the leg	97,5%	7. Good condition of dressing	92%		
8. Condensate in ventilatory circuits	24,2%	8. Urinary collecting bag below the level of bladder	100%	8. Chlorhexidine impragnated dressing	29%	4.Proportion of 5 movements for hand hygiene	After body fluid exposition risk: 66,5%
							Before aspetic task: 100%
							After patient contact: 76,3%
9. Gastric over- distention	8,6%	9. Urinary collecting bag with less than 75% of capacity full	95,25%	9.Type of set connector	Needle less connector (split-septum): 100%		After contact with patient surroundings: 60%
							Before patient contact: 53,5%
10. Stress ulcer prophylaxis	88,4%	10. Catheter indication:	Monitoring: 100%	10. Type of fluid container	Collapsible: 29% Semi- rigid: 71%	5.Stratified by time shift	Morning 80% Afternoon 64%
11. Deep vein thrombosis prophylaxis	Yes: 93% Contraindicated: 7%	11. Catheter type:	Indwelling: 100%	11. Administration equipment date	100%		
		12. Patients with inserted urinary catheter:	100%	12 Chlorhexidine washbathing	0%		

Abbreviations: *CA-UTI* catheter-associated urinary tract infection; *CLA-BSI* central line-associated bloodstream infection; *VAP* ventilator-associated pneumonia

## Discussion

In this study with prospective, continuous infections monitoring prevalence rate we found ICU acquired HAIs at 1/5 of patients. In a multicentre European Prevalence of Infection in Intensive Care (EPIC) study (1992) it was shown that infections were diagnosed in 4501 patients (48.8%) and ICU- acquired HAIs in 2064 patients among 10,038 patients (20.6%, nearly to our results) [29]. A pioneering study on infections at our ICU involving 560 patients hospitalised from 1995 to 1996 showed that HAIs affected 48% of patients, but ICU-acquired HAIs were diagnosed more frequently (33%) compared with this study [30]. The crude infection rate (prevalence of HAIs) analysed in our study was lower than in the previously published study carried out in our centre involving 847 patients treated from 2007 to 2010, in which they were 24.3% [9]. In the Polish point prevalence study (according to ECDC methodology) carried out in 2012–2013, the prevalence of HAIs in ICU patients was 430/945 (39.8%), significantly higher than in our study [15]. Moreover, the overall HAIs rate found during a ten-year study at the ICU in the District Hospital in Poland (27.6%) was also higher than our present result [6]. In the Polish multicenter study conducted 2013–2015 in seven Polish ICUs based on active surveillance the incidence of HAIs (22.6%) was similar to our results [16]. Reversely, the ECDC HAIs register analysed data from 2014 (on the basis The European Surveillance System –Tessy) showed that the HAIs incidence rate in 87,337 patients hospitalised > 24 h in 1290 European ICUs was twice lower (8%) than in our study [31]. In 2016 and 2017, according to the ECDC registers, the frequency of ICU acquired HAIs was on roughly the same level (8.4 and 8.3%) and still lower than our results [25, 31]. In a study conducted in the USA, the prevalence of HAIs in ICU patients was 34.5%, DA-HAIs (VAP, UTI,CLA-BSI) accounted for 25.6% of all HAIs [14]. Nevertheless, the rate of ICU acquired HAIs was not assessed [14]. The incidence density of HAIs in our study was lower than in the previously published study carried out in our centre (21.9/1000) [9]. The same study showed that the VAP incidence density was twice higher than the current results and the CLA-BSI incidence density was four times higher [9]. Incidence density of VAP in our study is lower than our earlier published results (11.15/9.34/10.23/1000) whereas incidence density of CA-UTI is still on the same high level (6.44/6.84/7.16/1000) [32, 33]. In other Polish observational study VAP (15.5/1000) and CLA-BSI (5/1000) incidence density was higher and CAUTI (1.9/1000) incidence density was lower than in our study [34]. Results of our study were similar to results of other studies when VAP was the most commonly diagnosed DA-HAI [6, 8, 10–12, 15]. Our results showed that clinical presentations of HAIs were more frequent than those found in European Centre Disease Control report (except for CLA-BSI), more frequent than the USA CDC report, yet less frequent than in limited-resource countries (except for CA-UTI) [10, 24, 25].

Our observations confirm that hospital infections considerably prolonged the LOS at ICU [9–11]. According to a multicenter worldwide study (2017) Prevalence and Outcomes of Infection among patients in ICUs (EPIC III) findings, median (IQR) length of ICU stay was 10(3–28) days whereas in infected patients it was 15(6–36), and 5(2–17) days in not infected [12]. LOS in our study in comparison to EPIC III findings was longer in regards to infected and nearly the same in not infected patients. The increase in the mortality rate due to DA-HAIs was shown in several studies [35–37]. In the first EPIC study the mortality rate in patients with infections (the comparison did not specify clinical form of DA-HAIs) was twice higher than in patients without infections (25% vs. 11%, *p* < 0.01) [29]. Higher mortality rate at infected patients (23.6%) than in not infected (9.6%) was found also in EPIC III study. However, this study did not specify mode of infection acquisition (community, ICU- acquired, hospital- acquired) in this analysis [12]. Another Polish study did not show, similarly to ours, any statistical difference in mortality in patients with and without infections (35.58% vs 39.23%, *p* = 0.5674) [38]. In recent years, an increase of GNB infections in hospital practice worldwide has been observed, which confirms the results of our study [12, 25, 31, 39]. Distribution of isolated microorganisms according to mode of acquisition infections in EPICIII study showed that at ICU-acquired infections GNB consisted 77.9%, GPB 31.3%, fungi 18.5%. In the same study infections caused by *Pseudomonas spp*.(23%), *Klebsiella spp*. (22.6%), *Acinetobacter spp*.(16,6%) were found more frequently [12]. Acinetobacter infections (independently from acquisition) were found more frequently in Asia/Middle East 25,6%, Eastern Europe 22.9%, Africa 15.8%, and very rarely in Western Europe 3,5%, Central/South America 9,4%, North America 1.0%, Australasia 1,9% [12]. Predominance of GNB in EPIC III study is similar to our study (77.9% vs 74.6%). The high percentage of *Acinetobacter baumannii* infections observed in our study is similar to that in hospitals in Greece and Italy and in another Polish centre, and is epidemic in its character [38, 40, 41]. Contrary to our findings, the most frequently isolated microorganism in ICU pneumonia episodes in Europe was *Pseudomonas spp* (19.9%), whereas *Acinetobacter spp.* reached only 4.5%. *Acinetobacter spp*. spreading at patients with ICU pneumonia varied and reached 39.5% in Romania, 20% Slovacia, whereas 0% in Belgium,1.5% in Germany, 1.8% in UK, 2.7% in France [25]. The European Antimicrobial Resistance Surveillance Network (EARS-NET) report from 2018 showed that *Acinetobacter spp* resistance to carbapenems was higher in Croatia 95.5%, Greece 92.4%, Romania 85.3%, Italy 79.2%, Poland 67.3%, Spain 54.3% and lower in Netherlands 4.6%, Germany 4.4%, Belgium 3.8%, Sweden 3.7%, UK 1.8% [42]. The EPIC III study found that infections caused vancomycin resistant Enterococcus, *Klebsiella pneumoniae* resistant to third generation cephalosporines and carbapenems, and carbapenem resistant *Acinetobacter spp*. was independently associated with a higher risk of death compared to other microorganisms [12]. Since *Acinetobacter baumannii* MDR/ carbapenem resistant infections (data not shown) were predominant in our patients, it may be the cause of such high mortality [43]. In regards to the last analysed elements of the study, it was found in several studies that HAIs preventive bundles are associated with DA-HAIs reduction [44, 45]. It has been demonstrated that implementation of specific INICC program directed to improve hand hygiene in limited resource countries improved HH compliance and contributed to the reduction DA-HAIs and mortality [46, 47]. Baseline HH compliance (64.66%) of ICU staff in our study was similar to earlier INICC data in which HH compliance rates were 9–75% [46]. Limitations of the study: firstly, because it is only one centre, study results can be different in relation to geographic regions and type of departments. Secondly, only compliance with bundle was assessed, not its influence on infection rate, given it was not the aim of the study. Thirdly, we did not assess and compare patients’ conditions in infection group and without infections using scoring methods, since it is not included in ISOS INICC methodology. Finally, numerous factors influence ICU LOS and hospital costs. Nevertheless, we showed an economic cost analysis using methods according to earlier INICC data, on a basis extra LOS and daily cost per patient [3, 4].

## Conclusions

Device associated hospital infections in our centre were found in nearly 1/5 of treated patients. VAP constituted more than half of those infections. DA-HAIs occurred more frequently than in the USA (except for CLA-BSI), yet less frequently than in the developing countries (except for CA-UTI). They prolonged the length of hospital stay and generated additional treatment costs. However, HAIs had no influence on the mortality rate. *Acinetobacter baumannii MDR* infections proved to be the most problematic therapeutic issue. Detailed registering of hospital infections using ISOS3, creating, implementing and monitoring compliance with hygienic and preventive measures, as well as the analysis of obtained data and drawing conclusions considered in hospital management may prove useful in improving the quality of care and reducing hospital costs.

## Data Availability

The data collected and analysed during this study are available and can be accessed from Wieslawa Duszynska (e-mail: wieslawa.duszynska@umed.wroc.pl).

## Abbreviations

CA-UTI: Catheter-associated urinary tract infection
CDC: Centre for Disease Prevention and Control
CI: Confidence interval
CL: Central line
CLA-BSI: Central line-associated bloodstream infection
CRP: C-reactive protein
DA-HAIs: Device-associated health care-associated infections
DU-R: Device utilisation ratio
ECDC: European Centre for Disease Prevention and Control
EPIC: European Prevalence of Infection in Intensive Care
€: Euro
EUCAST: European Committee on Antimicrobial Susceptibility Testing
GNB: Gram Negative Bacteria
GPB: Gram Positive Bacteria
HAIs: Health care-associated infections
HH: Hand Hygiene
HELICS: The Hospitals in Europe Link for Infection Control through Surveillance
ICU: Intensive Care Unit
IMA: INICC multidimensional approach
INICC: International Nosocomial Infection Control Consortium
ISOS: INNIC Surveillance Online System
IQR: Interquartile range
LOS: Length of stay
MDR: Multidrug-resistant
MRSE: Methicillin-resistant *Staphylococcus epidermidis*
MRCNS: Methicillin-resistant coagulase-negative staphylococcus
MV: Mechanical ventilator
NHSN: National Healthcare Safety Network
PCT: Procalcitonin
USA: The United States of America
UC: Urinary catheter
UH: University Hospital
US$: US dollar
VAP: Ventilator-associated pneumonia
